# Severe hyponatremia and diabetes insipidus caused by low-dose cyclophosphamide in breast cancer patients: A case report and literature review

**DOI:** 10.1097/MD.0000000000037572

**Published:** 2024-03-29

**Authors:** Yanfang Chen, Liye Li, Ruilian Ou, Yulan Wu, Baoying Pan, Linying Luo

**Affiliations:** a Department of General Surgery, The Third Affiliated Hospital of Guangzhou Medical University, Guangzhou, China; b Guangdong Provincial Key Laboratory of Major Obstetric Diseases, The Third Affiliated Hospital of Guangzhou Medical University, Guangzhou, China; c Guangdong Provincial Clinical Research Center for Obstetrics and Gynecology, The Third Affiliated Hospital of Guangzhou Medical University, Guangzhou, China.

**Keywords:** breast cancer, cyclophosphamide, diabetes insipidus, hyponatremia

## Abstract

**Rationale:**

Cyclophosphamide (CTX) is widely used in the treatment of malignancies and autoimmune diseases. Although severe hyponatremia caused by low-dose CTX chemotherapy is uncommon, it can lead to serious complications and even death.

**Patient concerns:**

A 44-year-old woman with left-sided breast cancer suddenly experienced headaches, disorientation and weakness after receiving low-dose neoadjuvant chemotherapy combined with CTX and doxorubicin.

**Diagnoses:**

The patient pathology showed invasive breast carcinoma. She developed severe hyponatremia and a generalized seizure after completing the first cycle of neoadjuvant chemotherapy with CTX and doxorubicin. Laboratory tests showed a serum sodium of 118 mmol/L (normal range 135–145 mmol/L) and potassium sodium 3.16 mmol/L (normal range 3.5–5.5 mmol/L). Subsequently, the patient developed secondary diabetes insipidus 4 hours after sodium supplementation, her 24-hour urine volume was 4730 mL (normal range 1000–2000 mL/24 hours), and the urine specific gravity decreased to 1.005.

**Interventions:**

The patient was given intravenous sodium chloride (500 mL of 3%NaCl, 100 mL/hour) and potassium chloride (500 mL of 0.3%KCl, 250 mL/hour). Meanwhile, she was advised to reduce her water intake, and pituitrin was administered to prevent dehydration caused by diabetes insipidus.

**Outcomes:**

The patient completely recovered after correcting of the serum sodium concentration (137 mmol/L) without any neurological deficits. After discontinuing pituitrin, her 24-hour urine volume was 2060 mL and the urine specific gravity was 1.015.

**Lessons:**

This is a typical case of severe hyponatremia induced by low-dose CTX. Clinicians and healthcare providers should be aware of this potential toxicity, and appropriate monitoring should be implemented.

## 1. Introduction

Cyclophosphamide (CTX) is a widely used antineoplastic drug that can be combined with other antineoplastic agents to treat various types of cancer, including breast, lymphoid, and pediatric malignancies.^[1–3]^ Although CTX is widely used to manage various diseases, there have been only a few reports of hyponatremia caused by it. Hyponatremia is defined as a serum sodium concentration lower than 135 mmol/L. It is the most common electrolyte abnormality in both inpatient and outpatient settings. Severe hyponatremia (serum sodium <120 mmol/L) is a medical emergency that can led to irreversible brain injury or death if not promptly treated.^[4]^ We report a case of severe symptomatic hyponatremia and diabetes insipidus that developed in a female breast cancer patient following the first cycle of chemotherapy containing low-dose CTX.

## 2. Case presentation

A 44-year-old woman presented to our hospital on January 17, 2023, with a left breast mass that had been present for more than 2 weeks. Her left breast had solid nodules measuring approximately 3.0 cm × 2.0 cm × 1.5 cm, which were classified as BI-RADS Class 4C after a breast ultrasound was performed. Histopathologic examination revealed infiltrating breast cancer, which was not a special type, with no estrogen receptor-positive cells, no progesterone receptor-positive cells, and no amplification of human epidermal growth factor receptor 2. However, 80% of the cells were positive for Ki-67. The patient was diagnosed with triple-negative breast cancer, which was staged as T2NxMx. The liver and kidney function, electrolyte levels, and other ancillary tests showed no abnormalities. There were no other medical issues in the patient history. Following extensive discussions with the patient, it was determined that initiating neoadjuvant chemotherapy would be the primary treatment approach.

**Figure 1.**
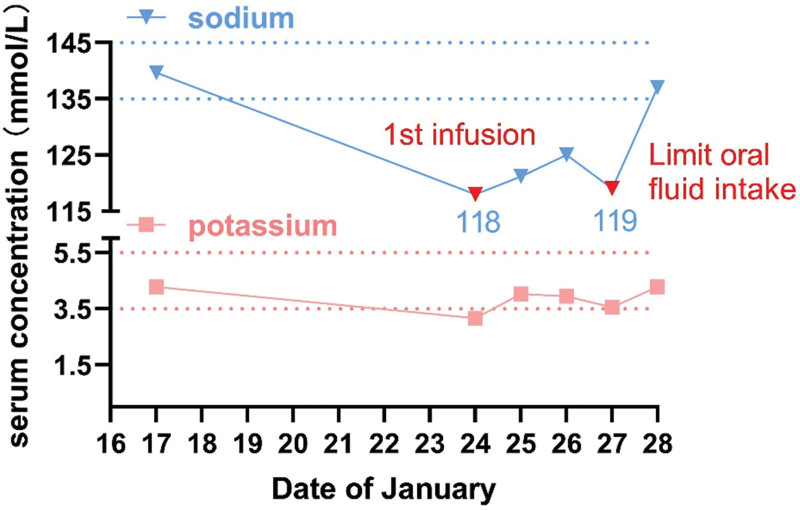
Time trend of serum sodium and potassium following the administration of low-dose CTX. CTX = cyclophosphamide.

On January 24, she received the first cycle of doxorubicin (100 mg/m^2^) and CTX (500 mg/m^2^) chemotherapy at 12:45. With supportive medications: 8 mg of dexamethasone, 5 mg of ondansetron, and 150 mg of aprepitant. During chemotherapy, the patient was administered 0.5 liters of isotonic saline for hydration and was advised to increase fluid intake to prevent hemorrhagic cystitis. The patient ingested approximately 2 liters of water after chemotherapy. At 17:00, the patient experienced palpitations, dizziness, and fatigue. Three hours later, the patient face appeared pale. Blood pressure was measured at 113/65 mm Hg, with a pulse rate of 68 beats per minute. ECG monitoring and low-flow oxygen (2 L/min) were used to improve the patient symptoms. At 20:00, the patient started experiencing delirium and speaking incoherently. An indwelling catheter was inserted to remove 800 mL of pale red urine. An emergency biochemistry examination showed a significant decrease in serum sodium levels from 138 to 118 mmol/L, and serum potassium levels from 4.28 to 3.16 mmol/L (Fig. 1). The patient was diagnosed with severe hyponatremia and immediately received intravenous sodium chloride (500 mL of 3% NaCl at a rate of 100 mL per hour). Subsequently, the patient was administered a potassium chloride injection (500 mL of 5% glucose with 10 mL of 15% potassium chloride) through intravenous drip.

**Figure 2.**
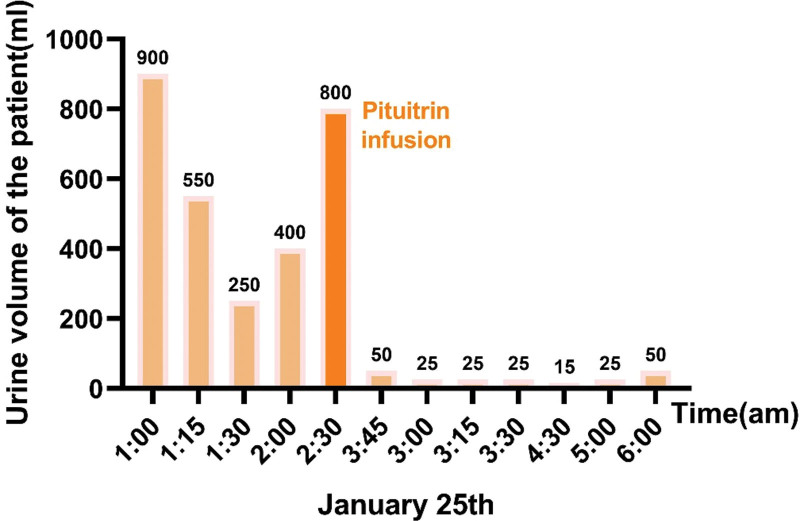
Time trend of patient urine volume following the administration of severe hyponatremia.

At 1:00 on January 25, the patient developed secondary diabetes insipidus after receiving sodium supplementation. Her 24-hour urine volume was 4730 mL (normal range: 1000–2000 mL/24 hours), with a urine output as fast as 1700 mL/h (01:00–02:00). Additionally, her urine specific gravity decreased to 1.005. An intravenous infusion of pituitrin was used to prevent dehydration caused by diabetes insipidus. Her serum sodium slowly rose to 121.2 mmol/L after 24 hours and 125 mmol/L after 48 hours (Fig. 1), and her mental status recovered. At the same time, the patient urine volume also returned to normal (Fig. 2). Diabetes insipidus has been effectively controlled. But on January 27, her serum sodium decreased to 119 mmol/L again without any neurological manifestations. After communicating with the patient, it was noted that she had consumed large quantities of water in a short time, which could have affected her electrolyte levels. She was advised to reduce her water intake. On January 28, 2023, the patient made a complete recovery and was discharged with a serum sodium level of 137 mmol/L and a serum potassium level of 3.98 mmol/L (Fig. 1). Three months later the patient underwent mastectomy for breast tumor in our hospital, the operation went smoothly, and after discharge the patient was followed up through WeChat, the patient did not have any discomfort, and the relevant indexes were all within the normal range.

## 3. Discussion

Hyponatremia caused by CTX can initially present with mild symptoms such as nausea, vomiting, weakness, headache, and dizziness. These symptoms may progress to more severe manifestations like irritability, lethargy, edema, confusion, and even loss of consciousness. In some cases, epileptiform seizures can also occur as severe symptoms. During CTX treatment, it is important to carefully monitor blood sodium levels and take prompt action to prevent further deterioration if patients experience any of these symptoms. To prevent further deterioration when patients develop the aforementioned symptoms, the clinical application of CTX should focus on monitoring blood sodium levels and taking timely action. When administered intravenously, hyponatremia typically occurs within 3 to 72 hours after administration.^[5–13]^ CTX-induced hyponatremia can be caused by either the initial dose or multiple doses.

Severe hyponatremia is a serious and potentially life-threatening condition.^[11]^ Early reported cases of severe hyponatremia were limited to patients treated with a single high-dose of CTX (30–40 mg/kg),^[14]^ and the incidence of severe hyponatremia with high-dose CTX has been reported as 5.8% in a retrospective study.^[15]^ However, with an increasing number of patients receiving CTX, moderate doses (20–30 mg/kg)^[16]^ and even low doses of CTX (<20 mg/kg)^[5–12]^ have been associated with severe hyponatremia. Symptomatic hyponatremia due to severe cyclophosphamide is very rare. The mechanism by which cyclophosphamide induces hyponatremia is unclear. However, it is believed that cyclophosphamide indirectly stimulates the release of antidiuretic hormone (ADH) and impairs the kidney ability to excrete water.^[7]^

In our study, the patient was administered CTX through intravenous infusion at a dose of 15 mg/kg. This resulted in severe hyponatremia, which was caused by the low-dose of CTX. The patient received hydration before and after administration of CTX to prevent hemorrhagic cystitis caused by the medication. Low serum sodium and potassium levels may be caused by an excess of water intake, leading to an overload of free water in the body. Physiologically, ADH is produced in the posterior pituitary gland in response to an increased plasma sodium concentration in order to conserve water. The syndrome of inappropriate antidiuretic hormone secretion is characterized by either a significant release of ADH in the absence of stimuli or by the increased action of ADH on the kidneys.^[3]^ Drug-induced syndrome of inappropriate antidiuretic hormone can occur due to either one or both mechanisms. The patient had normal electrolyte levels and renal function, and there was no evidence of neurological or endocrine abnormalities prior to treatment with CTX. It is reasonable to think that both CTX and the patient excessive water intake contributed to the severe hyponatremia. Interestingly, while correcting the patient hyponatremia, the patient developed acute diabetes insipidus. We believe this is a secondary effect of sodium supplementation. Specifically, the addition of high concentrations of sodium chloride inhibits the release of antidiuretic hormone, resulting in the rapid excretion of a significant amount of fluid that had been previously retained in the patient body. Fortunately, our medical team detected and intervened in time to prevent the patient from experiencing further electrolyte disturbances or dehydration. To the best of my knowledge, this is the first reported case of diabetes insipidus in the treatment of severe hyponatremia caused by low doses of cyclophosphamide. This should capture the attention of clinical workers.

Cyclophosphamide infusion can result in severe hyponatremia, which can cause serious neurologic toxicities and be life-threatening, although it is rare. It is crucial for physicians to be mindful of this side effect and treat it accordingly. Firstly, the prevention of hyponatremia associated with CTX relies on careful monitoring of blood electrolytes. This includes obtaining baseline data before administering the CTX and conducting regular post-administration checks to detect any changes. Secondly, it is essential to avoid excessive fluid intake and closely monitor the patient mental status when caring for patients undergoing low-dose CTX chemotherapy. Early detection of any abnormalities can be achieved through vigilant observation of the patient psychoneurological status, which allows for the timely implementation of refined nursing interventions. The Clinical Practice Guideline on Diagnosis and Treatment of Hyponatremia^[17]^ recommends the immediate intravenous administration of 3% sodium chloride solution in cases of severe hyponatremia. It also advises discontinuing the suspected medication.

## 4. Conclusion

In conclusion, our case highlights the rare occurrence of severe hyponatremia after administering low-dose CTX. Hypokalemia and secondary diabetes insipidus should also be considered in clinical practice when intravenous CTX is administered, particularly in patients with other underlying risk factors. In addition, healthcare providers should be aware of this potential toxicity, implement appropriate monitoring, and advise patients to notify their doctors if they experience any new or unusual symptoms.

## Acknowledgments

We would like to credit the patient for her participation in this case study.

## Author contributions

**Data curation:** Liye Li, Ruilian Ou, Linying Luo.

**Supervision:** Baoying Pan.

**Writing – original draft:** Yanfang Chen.

**Writing – review & editing:** Yulan Wu.
